# AStruct: detection of allele-specific RNA secondary structure in structuromic probing data

**DOI:** 10.1186/s12859-024-05704-x

**Published:** 2024-03-01

**Authors:** Qingru Xu, Xiaoqiong Bao, Zhuobin Lin, Lin Tang, Li-na He, Jian Ren, Zhixiang Zuo, Kunhua Hu

**Affiliations:** 1 State Key Laboratory of Oncology in South China, Cancer Center, Collaborative Innovation Center for Cancer Medicine, School of Life Sciences, Sun Yat-sen University, Guangzhou, 510060 China; 2 Department of Data Science, Dana-Farber Cancer Institute, Boston, MA 02215 USA; 3 Guangdong Key Laboratory of Liver Disease Research, The Third Affiliated Hospital of Sun Yat-sen University, Guangzhou, China

**Keywords:** Allele-specific events, RNA secondary structure, Structuromic probing data, Functional variants

## Abstract

**Background:**

Uncovering functional genetic variants from an allele-specific perspective is of paramount importance in advancing our understanding of gene regulation and genetic diseases. Recently, various allele-specific events, such as allele-specific gene expression, allele-specific methylation, and allele-specific binding, have been explored on a genome-wide scale due to the development of high-throughput sequencing methods. RNA secondary structure, which plays a crucial role in multiple RNA-associated processes like RNA modification, translation and splicing, has emerged as an essential focus of relevant research. However, tools to identify genetic variants associated with allele-specific RNA secondary structures are still lacking.

**Results:**

Here, we develop a computational tool called ‘AStruct’ that enables us to detect allele-specific RNA secondary structure (ASRS) from RT-stop based structuromic probing data. AStruct shows robust performance in both simulated datasets and public icSHAPE datasets. We reveal that single nucleotide polymorphisms (SNPs) with higher AStruct scores are enriched in coding regions and tend to be functional. These SNPs are highly conservative, have the potential to disrupt sites involved in m6A modification or protein binding, and are frequently associated with disease.

**Conclusions:**

AStruct is a tool dedicated to invoke allele-specific RNA secondary structure events at heterozygous SNPs in RT-stop based structuromic probing data. It utilizes allelic variants, base pairing and RT-stop information under different cell conditions to detect dynamic and functional ASRS. Compared to sequence-based tools, AStruct considers dynamic cell conditions and outperforms in detecting functional variants. AStruct is implemented in JAVA and is freely accessible at: https://github.com/canceromics/AStruct.

**Supplementary Information:**

The online version contains supplementary material available at 10.1186/s12859-024-05704-x.

## Introduction

Numerous genetic variants have been reported as risk or causative factors for diseases at the population level, as they disrupt DNA structures or regulatory elements and thereby impact transcription [1, 2]. There is increasing evidence that genetic variants can also affect RNA splicing [3], RNA secondary structure [4], RNA *N6*-methyladenosine (m6A) modification [5], and protein binding [6–8], etc. In individuals, these variants can act in an allele-specific manner, which are termed allele-specific events. Investigating the potential variants involved in allele-specific events is an important and effective approach to discovering and annotating functional variants.

RNA secondary structure is an essential feature that exerts a significant influence on several stages of the RNA life cycle, including RNA transcription [9], splicing [10] and translational control [11]. Facilitated by next-generation sequencing, a number of innovative techniques have been developed that combine traditional chemical probing methods with high-throughput sequencing to capture genome-wide RNA secondary structure. Foremost among them are reverse transcription stop (RT-stop) based structural probing techniques, including SHAPE-seq [12], icSHAPE [13] and smartSHAPE [14], etc.

Some genetic variants have been found to cause local or global RNA secondary structure changes [4] and have been proved to be associated with many genetic diseases [15, 16]. In the case of heterozygous variants, two alleles might have distinct effects on RNA secondary structure, namely allele-specific RNA secondary structure (ASRS). Several computational methods have been developed to predict ASRS without considering allele dosage [17–20]. However, analyzing ASRS remains a great challenge. Since the existing tools rely primarily on static sequence information, they are unable to capture the complex and dynamic cell conditions from the experimentally-derived data. Moreover, the RT-stop feature of sequencing technologies poses a challenge in effectively segregating reads by alleles and retaining sufficient structural information for analyzing the structure of each allele within a single sample.

To address the limitations mentioned above, we developed a computational tool called ‘AStruct’. To the best of our knowledge, AStruct is the first software capable of identifying allele-specific RNA secondary structure events at heterozygous single nucleotide polymorphisms (SNPs) within one sample using RT-stop based structuromic probing data.

## Methods

### Calculating allelic structure score from icSHAPE sequencing data

Six icSHAPE datasets of six human cell lines were downloaded from GEO [21] (Gene Expression Omnibus). Specifically, the K562, HepG2, HEK293, HeLa, and H9 datasets were obtained from GSE145805 [22], while the HEK293T dataset was obtained from GSE74353 [23]. smatSHAPE dataset of HEK293T was accessed through number GSE155961 [14]. Each dataset comprised two replicates of NAI-N3 treated samples and two replicates of control (DMSO) samples. The raw reads of each dataset were preprocessed using Trimmomatic [24] to remove barcodes and adapters. The preprocessed reads were then mapped to the human genome (GRCh38.p12) using STAR [25]. PCR duplicates were discarded by collapsing the reads with identical sequences.

673,668,919 human SNPs were download from NCBI SNP Database (dbSNP) (GCF000001405.38) [26]. Read coverage was calculated for each SNPs in each aligned sample. Only the heterozygous SNPs with reads covering both the reference (Ref) allele and the alternate (Alt) allele were retained. Moreover, the SNPs with total reads less than 10 or Ref reads less than 2 or Alt reads less than 2 were filtered out. For each retained SNP, a SNP window was determined by the start base of the most upstream SNP-spanning read and the end base of the most downstream SNP-spanning read. The structure score for each base in the window was calculated for Ref allele and Alt allele separately according to the following method modified from icSHAPE-pipe [27]:

Firstly, we calculated the scores for the RT-stop events and background in a SNP window using the reads overlapping the SNP window. Reads overlapping the SNP window but not spanning the SNP would be utilized for both Ref and Alt alleles. The first upstream base of a read mapping start represented an RT-stop event ($R$) and all the bases in a read represented the background ($B$). Accordingly, $R$ score and $B$ score were calculated for each base in the window using all reads.

Secondly, to smooth the $R$ scores and $B$ scores, all the values were divided by a normalization factor which was the 95th percentile of all scores in the window (see formulas 1 and 2).

1 $$R_{i} = R_{i} /{\varvec{R}}\left[ {0.95 \times N} \right]$$

2 $$B_{i} = B_{i} /{\varvec{B}}\left[ {0.95 \times N} \right]$$

where ${\varvec{R}}$ were arranged in ascending order as $R_{1} \le R_{2} \le \ldots \le R_{N}$ representing the RT-stop event occurring in all bases, ${\varvec{B}}$ were arranged in ascending order as $B_{1} \le B_{2} \le \ldots \le B_{N}$ representing the background in all bases, $i$ was the $ith$ base in the window, and $N$ was the window length.

Thirdly, an enrichment score ($ES$) was calculated for each base in the window using the smoothed $R$ scores and $B$ scores in NAI-N_3_ treated samples and DMSO control samples (see formula 3).

3 $$ES_{i} = {\raise0.7ex\hbox{${\left( {R_{i}^{T} - s \times R_{i}^{C} } \right)}$} \!\mathord{\left/ {\vphantom {{\left( {R_{i}^{T} - s \times R_{i}^{C} } \right)} {B_{i}^{C} }}}\right.\kern-0pt} \!\lower0.7ex\hbox{${B_{i}^{C} }$}}$$

where $ES_{i}$ was the enrichment score for the $ith$ base in the window, $R_{i}^{T}$ was the $R$ score for the $ith$ base in the window in treated sample, $R_{i}^{C}$ was the $R$ score for the $ith$ base in the window in control sample, $B_{i}^{C}$ was the $B$ score for the $ith$ base in the window in control sample, and $s$ was the predefined factor.

Fourthly, the enrichment score was converted between 0 and 1 using formula 4 to represent the structure score.

4 $$ES_{i} = \max \left( {0,\min \left( {1,\frac{{ES_{i} - {\varvec{ES}}\left[ {0.05 \times N} \right]}}{{{\varvec{ES}}\left[ {0.95 \times N} \right] - {\varvec{ES}} \left[ {0.05 \times N} \right]}}} \right)} \right)$$

where ${\varvec{ES}}$ were arranged in ascending order as $ES_{1} \le ES_{2} \le \ldots \le ES_{N}$.

Finally, we obtained a set of structure scores for *Ref* allele and *Alt* allele, separately.

### Statistical test for the structure difference between Ref allele and Alt allele

To test the structure difference between *Ref* allele and *Alt* allele, we firstly calculated *Pearson Correlation* between the structure scores of two alleles. Then the *Pearson Correlation Coefficient* was converted to the experimental structural disruption coefficient ($eSDC$) as described below [28] (see formula 5).

5 $$eSDC = \left( {1 - ^{p} CC} \right) \times \surd N$$

where $^{p} CC$ was the *Pearson Correlation Coefficient* of the structure scores between two alleles, and $N$ was the length of the SNP window. The larger $eSDC$ meant more difference in structure scores between the two alleles.

To evaluate the statistical significance of the difference, 1000 permutations were performed by assigning reads to *Ref* and *Alt* alleles following 1:1 ratio based on *Poisson distribution*. The *P* value of the difference was calculated by comparing the true $eSDCs$ and permuted $eSDCs$. Some studies have shown the robustness of measuring the gene expression difference by combining the magnitude and significance [29]. Thus, borrowing from this idea, we also combined $eSDC$ (magnitude) and *P* (statistical significance) to measure the structure difference. Because the reads that overlapping with the SNP window but not spanning SNP smoothed the structure difference between two alleles, we used the ratio between the number of reads spanning SNP and total reads in the window to adjust the difference score. Finally, the AStruct score was defined as formula 6:

6 $$AStruct\;score = - \log_{10} P \times eSDC \times \left( {1 - \log_{10} \left( {\frac{{R_{d} }}{R}} \right)} \right)$$

where ${\varvec{R}}_{{\varvec{d}}}$ was the number of reads spanning SNP, ${\varvec{R}}$ was the number of total reads in the SNP window.

### Calculating the structure difference for each base in a SNP window

To provide more details of the structure difference between the two alleles for each base, we applied the method from a previous study [30] as described below (see formula 7):

7 $$StrucDiff_{i} = \mathop \sum \limits_{k = i - 2}^{k = i + 2} {\raise0.7ex\hbox{${abs\left( {ES_{k,Alt} - ES_{{k,{\text{Re}} f}} } \right)}$} \!\mathord{\left/ {\vphantom {{abs\left( {ES_{k,Alt} - ES_{{k,{\text{Re}} f}} } \right)} 5}}\right.\kern-0pt} \!\lower0.7ex\hbox{$5$}}$$

A sequence of *P* values named $StrucDiff$ for each base were obtained by permutation test.

### Performance evaluation using simulated datasets

To evaluate the reliability of our method, we simulated the icSHAPE reads under different read depths (10M, 20M,…,100M) based on *Poisson distribution*. The pair or unpair structure information were generated from RNAsubopt [31]. In order to simulate the RT-stop event, the artificial stop intervention was evenly added on the unpaired bases. For the sequences without allelic structure difference, *Ref* and *Alt* alleles were designed to have the same set of unpaired bases (RT-stop bases). For the sequences with allelic structure difference, *Ref* and *Alt* alleles were supposed to have different sets of unpaired bases.

### Functional annotation of allele specific RNA secondary structure

All the retained SNP sites were divided into three groups according to the magnitude of AStruct score, which are ‘Low’ (0), ‘Medium’ (0, 1], and ‘High’ (1, + ∞). For RNAsnp, the ‘High-RNAsnp’ and ‘Low-RNAsnp’ groups were defined by smallest 10% *r* and largest 10% *r* values of the same SNP sites [20]. The *r* value represented the *Pearson Correlation Coefficient* for the structural comparison between the reference sequence and the alternative sequence (± 100bps of SNP sites). SNPs in linkage disequilibrium (LD) were retrieved from HaploReg (*r*^2^ > 0.8) [32].

To explore conservation, we gathered PhastCon100 scores from UCSC genome browser [33]. In examining the relationship between ASRS and other AS events, genetic variants affecting RNA modifications (mainly m6Asnp) were obtained from RMVar [34]. Allele-specific RBP binding (ASRBP) were collected from ADASTRA [6] overlapped with two RBP datasets [35, 36]. To investigate ASRS association with diseases, FATHMM-XF scores were annotated to all SNPs [37]. Disease-related SNPs were obtained from ClinVar [38].

## Results

### AStruct pipeline for evaluating allele-specific structure events

**Fig. 1:**
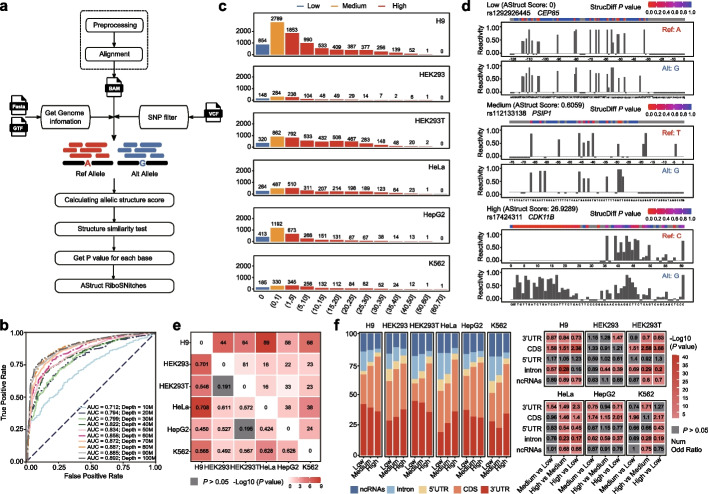
Robustness of AStruct in predicting allele-specific structure events. **a** Flowchart of AStruct pipeline. **b** ROC curves of simulated datasets under different sequencing depths. **c** The AStruct score distribution of all heterozygous SNPs in six cell lines (H9, HEK293, HEK293T, HeLa, HepG2, and K562). **d** The allele level reactivity score derived from the icSHAPE data of K562 cell line for three SNPs representing the three AStruct group (Low: rs1292926445; Medium: rs12133138; High: rs17424311). The *P* values indicating the difference in reactivity score between two alleles for each base were shown in the top. **e** The correlation of ASRS SNPs between different cell lines. The value in top-right triangle presents the number of common ASRS SNPs; the value in bottom-left triangle presents the *Pearson correlation coefficient* of the AStruct scores. Background color presents the *P* Value, where gray indicates *P* > 0.05. **f** Distribution of ASRS SNPs in different genomic regions (3′UTR, CDS, 5′UTR, intron, and ncRNAs). Left: Stacked bar plot for three AStruct groups. Right: Comparison the sites number of different regions between different groups (*Fisher’s exact test*)*.* The number represents the Odds Ratio. Background color represents the significant *P* Value

AStruct is implemented in Java (JDK 8), and simply takes sorted BAM files from structure sequencing data as input. The rationale behind AStruct is that the two alleles involved in an allele-specific structure event are expected to have dissimilar structure scores using structure sequencing technologies such as icSHAPE and smartSHAPE [13, 14]. The workflow of AStruct was illustrated in Fig. 1a. First, the preprocessed sequencing reads were aligned to the reference genome using STAR [25]. Second, all the SNPs obtained from dbSNP [26] were used to search against the alignment data, and only the heterozygous SNPs were retained for further analysis. Third, the aligned reads around each heterozygous SNP were separated into two groups according to the SNP, with each group representing one allele. Finally, the AStruct score for each SNP was calculated based on the similarity test between the structure scores of two alleles. A higher AStruct score indicated higher allele specificity of RNA secondary structure.

### AStruct is robust in predicting allele-specific structure events

To evaluate the performance of AStruct, we simulated 10 icSHAPE datasets with sequencing depths ranging from 10 to 100M. Each dataset consisted of two control samples and two treated samples. The AUC values of the simulated datasets ranged from 0.712 to 0.892, indicating a robust performance of AStruct. As expected, the performance of AStruct improved with the increase of sequencing depth (Fig. 1b).

We next applied AStruct to public icSHAPE datasets of six different cell lines. As a result, we obtained 1616, 3046, 930, 4415, 2591, and 8640 heterozygous SNPs with enough reads coverage and enough extra reads stop of structure information from K562, HepG2, HEK293, HEK293T, HeLa, and H9, respectively (Fig. 1c). According to the AStruct score, the heterozygous SNPs above were categorized into three groups: the ‘Low’ group with a score of 0, the ‘Medium’ group with a score ranging from 0 to 1, and the ‘High’ group with a score greater than 1. We applied a published method [30] to measure the structure difference at the single base level. As shown in Fig. 1d, the ‘High’ group had much more bases with significantly different structure scores between two alleles, compared to the ‘Low’ group and ‘Medium’ groups. We found a high correlation between AStruct scores of different cell lines (Fig. 1e), further demonstrating the robustness of AStruct. Additionally, we found that SNPs with higher AStruct scores were more likely to be located in the CDS region rather than the intron region (Fig. 1f). Moreover, AStruct could also be applied to smartSHAPE, another RT-stop based sequencing technology, implying broader applicability (Additional file 1: Fig. 1).

### AStruct can help identify functional variants

Numerous causal SNPs associated with traits or diseases were defined in previous GWAS studies, but most of them were usually not genotyped but are in linkage disequilibrium (LD) with the genotyped SNPs [39]. Recent studies on SNP pairs in high LD indicated their interplay on m^6^A modification [40] and RNA structure [41]. Consequently, in order to take the effects of LD-SNPs into account, we annotated the LD-SNPs (*r*^2^ > 0.8) for ASRS SNPs from HaploReg [8] and grouped ASRS SNPs with their LD-SNPs into new ‘High’, ‘Medium’ and ‘Low’ groups for the following functionality analyses. We presumed that SNPs in the ‘High’ group are prone to include allele-specific structure features than SNPs in the ‘Medium’ or ‘Low’ groups.

**Fig. 2:**
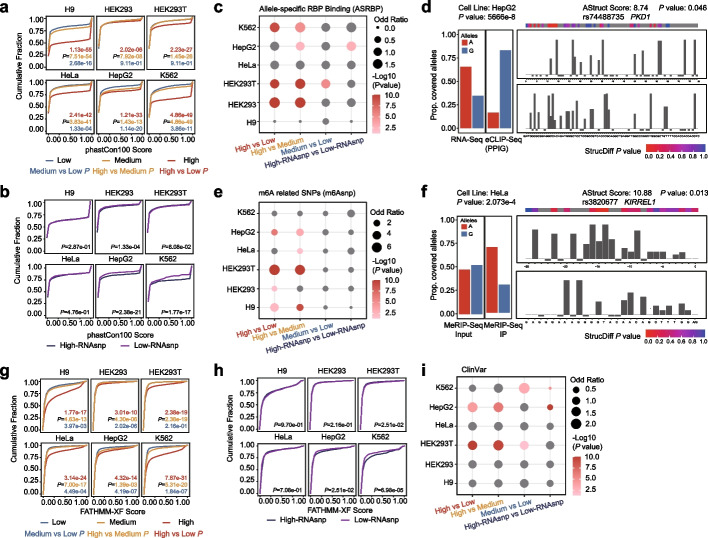
AStruct is valuable in assisting with the identification of functional variants. **a**, **b** The cumulative distribution curve of the phastCon100 score for different groups. a for AStrcut, b for RNAsnp. **c** The bubble plot shows the comparison of the number of ASRS SNPs annotated as allele-specific RBP binding events between different groups. **d** A G-to-A SNP (rs74488735) in the ‘High’ group shows allele-specific pattern of a PPIG binding site in PXD1 of HepG2 cell line. It is illustrated by the different proportion of reads count covering each allele in the eCLIP-Seq data and the control RNA-Seq data (left), and the significant structure difference with each allele (right). **e** The bubble plot shows the comparison of the number of ASRS SNPs annotated as m6A related variants between different groups. **f** A A-to-G SNP (rs3820677) in the ‘High’ group shows allele-specific pattern of a m6A modification site in KIRREL1 of HeLa cell line. It is illustrated by the different proportion of reads count covering each allele in the MeRIP-Seq IP data and the control Input data (left), and the significant structure difference with each allele (right). **g**, **h** The cumulative distribution curve of the FATHMM-XF score for different groups. **g** For AStrcut, h for RNAsnp. **i** The bubble plot shows the comparison of the number of disease-related ASRS SNPs annotated by ClinVar between different groups. The *P* value of cumulative distribution curve was calculated using *Kolmogorov–Smirnov (KS) test*. The *P* value of bubble plot was calculated using *Fisher’s exact test*

As shown in Fig. 2a, SNPs with higher AStruct scores illustrated a higher degree of conservation, highlighting their important function. As for the same test on RNAsnp, which predicted ASRS based on sequence, only three cell lines showed significant conservation differences (Fig. 2b). This observation led us to concentrate on investigating the biological features associated with ASRS.

ASRS SNPs with high AStruct scores demonstrated an enrichment of allele-specific RBP binding sites (ASRBP) and m6A related SNPs (m6Asnp) compared to SNPs with medium or low scores. Again, we did not detect any significant differences between ‘High-RNAsnp’ and ‘Low-RNAsnp’ groups (Fig. 2c and 2e). Besides, we found 39 original ASRS SNPs with high AStruct scores in HepG2 cell line that can induce allele-specific binding pattern of the PPIG RBP (Additional file 1: Table 1). For example, the SNP rs74488735 located in *PKD1* showed both allele-specific RNA secondary structure (AStruct score = 8.74, *P* = 0.046) and allele-specific PPIG RNA binding pattern (*P* = 5.666e−8) (Fig. 2d). Moreover, we found 34 original ASRS SNPs with high AStruct scores in HeLa cell line that can influence the m6A modification (Additional file 1: Table 2). For instance, the SNP rs3820677 located in *KIRREL1* showed both allele-specific RNA secondary structure (AStruct score = 10.88, *P* = 0.013) and allele-specific m6A modification (*P* = 2.073e−4) (Fig. 2f).

Furthermore, to investigate the association between ASRS SNPs and disease, the FATHMM-XF score was used to evaluate the pathogenicity [37]. A higher FATHMM-XF score means mutations are more likely to be pathogenic, while a lower score means more benign. We found that SNPs with higher AStruct scores were more pathogenic (Fig. 2g) while RNAsnp did not reveal any significant differences [20] between different groups (Fig. 2h). We also annotated the ASRS SNPs and their LD-SNPs using ClinVar database [38], and found SNPs with higher AStruct scores had a higher proportion of variants associated with disease, such as Lynch syndrome, Argininosuccinate lyase deficiency, and Farber disease (Fig. 2i, Additional file 2: Table 3).

Taken together, AStruct demonstrated superior capability in detecting functional ASRS SNPs, in contrast to tools that only focused on static sequence information. ASRS SNPs with high AStruct scores tended to be more conserved and were more likely to influence other allele-specific events such as RNA–protein interactions and RNA modifications. These ASRS SNPs are also potentially linked with specific traits- or diseases-related variants.

## Discussion

Despite the fact that the impact of genetic variants on RNA structure and transcriptional regulation were widely acknowledged, specialized tools to systematically profile ASRS have been lacking. Identification of ASRS events is expected to contribute to revealing functional variants and illuminating the molecular mechanism of associated diseases at the allele level. Here, we provided AStruct as a robust algorithm for detecting ASRS from RT-stop based structuromic probing data.

Theoretically, RNAsnp and other sequence-based tools are designed to evaluate the impact of SNPs on local RNA secondary structure. They typically utilize thermodynamic models on the input sequence. However, the structures and functions are mostly varied because of cell specificity, cell status and their microenvironment that must be considered. Taking full advantage of experimental structure sequencing technologies, AStruct utilized allelic variants, base pairing, and RT-stop information under in vivo conditions to detect dynamic and functional ASRS. In practice, we analyzed the association of ASRS ANPs detected from AStruct and RNAsnp with other AS events as well as diseases. The results further demonstrated the superiority of AStruct. Additional tests on smartSHAPE further indicated the broad applicability of AStruct.

However, it is worth noting that read coverage is important for recovering RNA structure and providing enough SNPs coverage for statistical analyses. In this study, we set coverage threshold for each SNPs. Because of the sparsity of alleles and the truncation in RT-stop data, only a part of SNPs satisfied the requirements and were used as candidates. Therefore, a complete mapping of ASRS have not yet been established. These limitations can be overcome by using high sequencing depth data and high resolution technologies. Moreover, we are working to update AStruct to adapt it to RT-Mut structure sequencing data. While not mentioned in this article, we had successfully tested AStruct on SHAPE-MaP datasets and obtained similar results. It is expected that AStruct can be applied to more types of structure sequencing data and provide insights to allele-specific RNA secondary structure.

## Conclusions

In conclusion, AStruct can be an excellent candidate for detecting allele-specific RNA secondary structure. The advantages of AStruct are obvious. Using high-throughput RT-stop based experimental data, AStruct has the capability to capture the allele-specific RNA secondary structure in the real cellular environment. In addition, AStruct allows allele-specific comparisons within a single sample by accurately separating alleles based on heterozygous SNPs, resulting in more accurate and reliable results.

## Availability and requirements

Project name: AStruct. Project homepage: https://github.com/canceromics/AStruct. Operating system: Platform independent. Programming language: Java. Other requirements: JDK 8. License: GNU GPL v3. Any restrictions to use by non-academics: none.

### Supplementary Information

**Additional file 1: Fig. 1.** AStruct in smartSHAPE. **a** Venn diagram of icSHAPE and smartSHAPE results. Pearson Correlation was calculated using the AStruct scores of the intersection set. **b** The cumulative distribution curve of the FATHMM-XF score for three AStruct groups identified using smartSHAPE. **Table 1.** ASRS SNPs in ‘High’ group result in allele-specific RNA-protein-interaction of PPIG protein in HepG2 cell line. **Table 2.** ASRS SNPs in ‘High’ group result in allele-specific m6A modification in HeLa cell line.

**Additional file 2: Table 3.** Disease annotation for ASRS SNPs and LD-SNPs.

## Data Availability

Public datasets used in this article are available under GEO accession number GSE145805, GSE74353 (icSHAPE), GSE155961 (smartSHAPE), GSE198145 (RNA Seq), GSE91478 (CLIP Seq), and GSE102493 (MeRIP Seq).

## Abbreviations

AS: Allele-specific
ASE: Allele-specific gene expression
ASM: Allele-specific methylation
ASB: Allele-specific binding
ASRS: Allele-specific RNA secondary structure
ASRBP: Allele-specific RNA-binding protein
RBP: RNA-binding protein
RT: Reverse transcription
PCR: Polymerase chain reaction
SHAPE-seq: Selective 2′-hydroxyl acylation analyzed by primer extension and sequencing
icSHAPE: In vivo click selective 2-hydroxyl acylation and profiling experiment
smartSHAPE: Small amount random RT icSHAPE
NAI-N3: 2-Methylnicotinic acid imidazolide, by the addition of an azide to the nicotinic acid ring
DMSO: Dimethylsulfoxide
SNP: Single nucleotide polymorphism
m^6^A: N6-methyladenine modification
Ref: Reference
Alt: Alternate
ES: Enrichment score
eSDC: Experimental structural disruption coefficient
LD: Linkage disequilibrium
