# Supplementary material for: AStruct: detection of allele-specific RNA secondary structure in structuromic probing data
Source: BMC Bioinformatics. 2024 Mar 1;25:91. doi: 10.1186/s12859-024-05704-x (PMC11264973; doi:10.1186/s12859-024-05704-x)
Supplement: Supplementary file 1 — Additional file 1: Fig. 1. AStruct in smartSHAPE. a Venn diagram of icSHAPE and smartSHAPE results. Pearson Correlation was calculated using the AStruct scores of the intersection set. b The cumulative distribution curve of the FATHMM-XF score for three AStruct groups identified using smartSHAPE. Table 1. ASRS SNPs in ‘High’ group result in allele-specific RNA-protein-interaction of PPIG protein in HepG2 cell line. Table 2. ASRS SNPs in ‘High’ group result in allele-specific m6A modification in HeLa cell line. [file 12859_2024_5704_MOESM1_ESM.docx]

**Supplementary Figure**


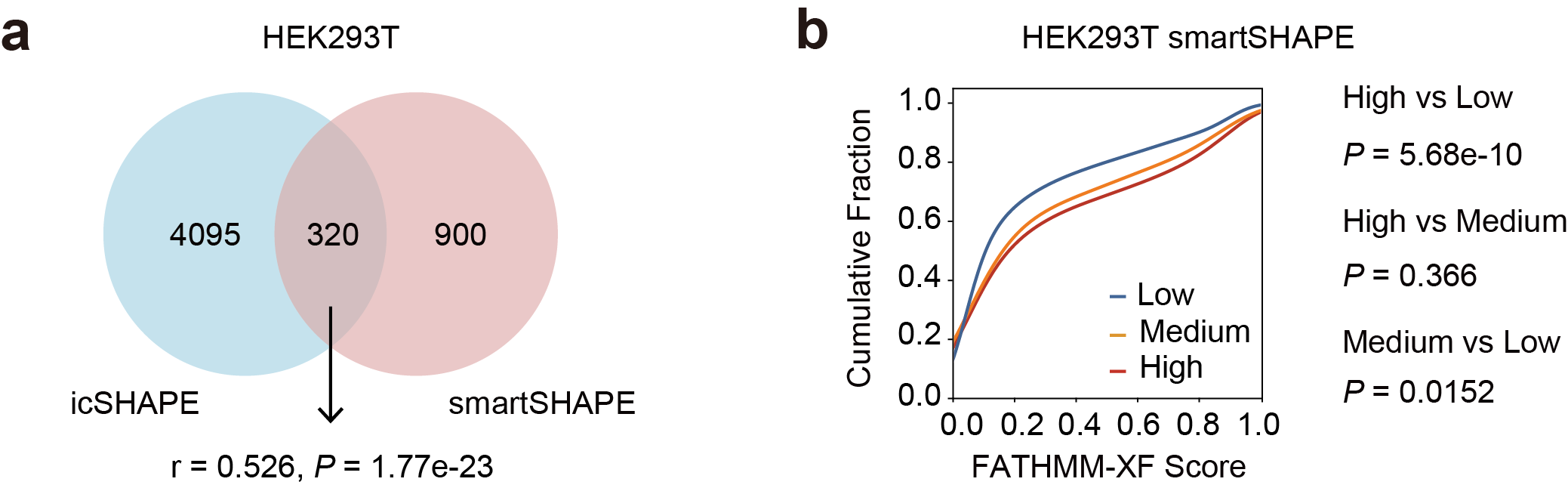


**Supplementary Figure 1. AStruct in smartSHAPE.** **a,** Venn diagram of icSHAPE and smartSHAPE results. Pearson Correlation was calculated using the AStruct scores of the intersection set. **b,** The cumulative distribution curve of the FATHMM-XF score for three AStruct groups identified using smartSHAPE.

**Supplementary Table**

**Supplementary Table 1** ASRS SNPs in ‘High’ group result in allele-specific RNA-protein-interaction of PPIG protein in HepG2 cell line.

| **Chr** | **Position** | **Ref** | **Alt** | **rsID** | **Gene** | **eCLIP Counts** | **RNA-Seq Counts** | **P Value** |
| --- | --- | --- | --- | --- | --- | --- | --- | --- |
| chr1 | 11840380 | T | C | rs198412 | CLCN6 | 15\|6 | 7\|14 | 2.937e-2 |
| chr1 | 21852751 | C | T | rs35669711 | HSPG2 | 17\|2 | 10\|12 | 3.763e-3 |
| chr1 | 43619068 | C | T | rs1143701 | PTPRF | 9\|14 | 78\|46 | 3.941e-2 |
| chr1 | 43981741 | G | C | rs1859728 | B4GALT2 | 31\|2 | 104\|57 | 6.404e-4 |
| chr2 | 27238100 | A | G | rs1141313 | CAD | 4\|30 | 39\|26 | 4.065e-6 |
| chr2 | 55617309 | T | C | rs782594 | PPP4R3B | 12\|2 | 12\|33 | 1.277e-4 |
| chr2 | 131516901 | G | A | rs13010688 | LOC150776 | 56\|10 | 36\|24 | 2.382e-3 |
| chr2 | 207624377 | C | A | rs4234080 | METTL21A | 13\|3 | 43\|56 | 6.355e-3 |
| chr2 | 238238715 | G | A | rs77852620 | HES6 | 2\|18 | 120\|202 | 1.456e-2 |
| chr3 | 48415297 | G | A | rs765476248 | PLXNB1 | 22\|43 | 65\|60 | 2.129e-2 |
| chr3 | 191369951 | A | T | rs35380043 | CCDC50 | 12\|21 | 69\|52 | 4.857e-2 |
| chr5 | 146013801 | C | T | rs758037 | SH3RF2 | 11\|3 | 8\|12 | 3.818e-2 |
| chr6 | 130869698 | G | A | rs17059736 | EPB41L2 | 32\|2 | 29\|9 | 4.992e-2 |
| chr7 | 5308283 | G | A | rs11554710 | TNRC18 | 2\|14 | 30\|28 | 8.807e-3 |
| chr7 | 100149507 | G | A | rs12878 | LAMTOR4 | 18\|10 | 95\|136 | 2.575e-2 |
| chr8 | 18071302 | C | T | rs1071645 | ASAH1 | 6\|17 | 78\|52 | 3.086e-3 |
| chr8 | 94529074 | A | G | rs957448 | VIRMA | 23\|4 | 4\|6 | 1.163e-2 |
| chr9 | 17727 | G | A | rs771704615 | WASHC1 | 66\|10 | 201\|80 | 6.962e-3 |
| chr10 | 27186515 | T | C | rs10741130 | MASTL | 6\|3 | 11\|39 | 1.272e-2 |
| chr10 | 92689976 | G | A | rs117639730 | HHEX | 3\|8 | 9\|2 | 2.997e-2 |
| chr12 | 102917201 | T | G | rs2280615 | PAH | 6\|4 | 10\|42 | 1.390e-2 |
| chr12 | 132659414 | C | T | rs5744857 | POLE | 16\|34 | 23\|17 | 1.925e-2 |
| chr13 | 72775221 | G | C | rs7332388 | DIS3 | 16\|8 | 5\|14 | 1.390e-2 |
| chr16 | 2114210 | G | A | rs74488735 | PKD1 | 253\|51 | 10\|19 | 5.666e-8 |
| chr16 | 67657765 | A | G | rs6979 | ACD | 5\|2 | 59\|124 | 4.421e-2 |
| chr16 | 68356794 | C | T | rs61733486 | PRMT7 | 27\|21 | 79\|171 | 1.619e-3 |
| chr16 | 70269677 | G | A | rs2070203 | AARS | 25\|23 | 38\|93 | 7.583e-3 |
| chr16 | 88652144 | G | A | rs11076693 | MVD | 41\|35 | 416\|178 | 5.897e-3 |
| chr16 | 88713331 | A | G | rs11549837 | CTU2 | 4\|20 | 67\|33 | 1.391e-5 |
| chr16 | 88725050 | G | A | rs11645197 | PIEZO1 | 47\|23 | 29\|49 | 3.053e-4 |
| chr17 | 1474649 | A | G | rs2286873 | MYO1C | 19\|9 | 12\|22 | 2.086e-2 |

**Supplementary Table 2** ASRS SNPs in ‘High’ group result in allele-specific m6A modification in HeLa cell line.

| **Chr** | **Position** | **Ref** | **Alt** | **rsID** | **Gene** | **MeRIP IP**  **Counts** | **MeRIP Input Counts** | **P Value** |
| --- | --- | --- | --- | --- | --- | --- | --- | --- |
| chr1 | 15429559 | T | C | rs1329757295 | EFHD2 | 856\|2 | 474\|9 | 2.476e-3 |
| chr1 | 158098554 | A | G | rs3820677 | KIRREL1 | 127\|54 | 48\|53 | 2.073e-4 |
| chr1 | 175989442 | T | C | rs111531372 | COP1 | 8\|42 | 78\|117 | 1.450e-3 |
| chr3 | 9384260 | G | C | rs1046788 | THUMPD3 | 107\|110 | 322\|184 | 3.785e-4 |
| chr5 | 177090990 | T | C | rs45526036 | FGFR4 | 88\|19 | 63\|39 | 1.137e-3 |
| chr7 | 6153890 | G | C | rs61729726 | USP42 | 8\|16 | 49\|32 | 2.174e-2 |
| chr7 | 150336793 | A | G | rs3735171 | LRRC61 | 196\|867 | 21\|155 | 4.134e-2 |
| chr8 | 19458575 | G | A | rs12155539 | CSGALNACT1 | 243\|62 | 256\|24 | 5.967e-5 |
| chr8 | 23144475 | A | G | rs1133782 | TNFRSF10D | 27\|97 | 6\|72 | 1.033e-2 |
| chr9 | 93476296 | C | T | rs10821135 | FAM120A | 192\|425 | 181\|676 | 1.575e-5 |
| chr9 | 137716669 | A | T | rs76684726 | EHMT1 | 1193\|203 | 351\|94 | 1.422e-3 |
| chr11 | 20508346 | A | G | rs74772442 | PRMT3 | 124\|24 | 181\|82 | 9.515e-4 |
| chr11 | 27340812 | T | G | rs17244028 | CCDC34 | 1040\|695 | 259\|219 | 2.419e-2 |
| chr11 | 32103432 | T | C | rs4876 | RCN1 | 3759\|2271 | 3895\|2161 | 2.469e-2 |
| chr11 | 47183430 | G | C | rs901747 | PACSIN3 | 2\|11 | 4\|2 | 4.603e-2 |
| chr11 | 61800254 | T | C | rs1127959 | FADS1 | 315\|6 | 207\|11 | 4.581e-2 |
| chr11 | 62532473 | A | C | rs11231130 | AHNAK | 215\|326 | 114\|126 | 4.950e-2 |
| chr11 | 68903951 | C | T | rs4930624 | IGHMBP2 | 35\|36 | 10\|28 | 2.510e-2 |
| chr11 | 69667426 | C | T | rs116600405 | ORAOV1 | 16\|60 | 24\|16 | 6.586e-5 |
| chr12 | 53041211 | C | G | rs1961466 | EIF4B | 446\|227 | 511\|363 | 1.839e-3 |
| chr12 | 56242441 | T | G | rs1592385681 | ANKRD52 | 243\|6 | 140\|14 | 3.919e-3 |
| chr14 | 60165381 | T | A | rs568729153 | DHRS7 | 2\|12 | 5\|2 | 1.729e-2 |
| chr14 | 91947643 | G | A | rs7149187 | FBLN5 | 18\|7 | 14\|28 | 2.725e-3 |
| chr15 | 40335644 | G | A | rs55641696 | CCDC9B | 117\|108 | 40\|14 | 3.593e-3 |
| chr15 | 41523318 | G | C | rs8027526 | RPAP1 | 49\|111 | 62\|74 | 1.126e-2 |
| chr15 | 43769604 | C | T | rs1053492 | PDIA3 | 1713\|725 | 1336\|656 | 2.254e-2 |
